# Self-Detecting Traffic Interference Control for Multi-Zone Services under 5G-Based Cellular Networks

**DOI:** 10.3390/s21072409

**Published:** 2021-03-31

**Authors:** Chongdeuk Lee

**Affiliations:** Division of Electronic Engineering, Jeonbuk National University, Jeonbuk 54896, Korea; cdlee1008@jbnu.ac.kr

**Keywords:** D2D, 5G cellular networks, real-time traffic, C2D communication, traffic interference

## Abstract

In this paper, we propose a multi-zone service control scheme to maximize the performance of each service zone when a large number of cellular service zones and Device-to-Device (D2D) service zones are composed into the 5G cellular network. This paper also improves performance of service zone by dividing traffic into real-time traffic and non-real-time traffic in order to minimize traffic interference. Real-time traffic and non-real-time traffic have a significant impact on communication performance. We propose a new self-detection traffic interference control technique to improve the Quality of Service (QoS) and throughput of D2D and Cellular-to-Device (C2D) communication in a cellular network, Self-detecting Traffic Interference Control Scheme (STICS). The proposed STICS mechanism distinguishes between short-term traffic congestion process and long-term traffic congestion process according to traffic characteristics to detect and control traffic. When the proposed scheme is applied to the 5G-based cellular network environment, it is expected that the traffic type will be efficiently classified by self-detecting the traffic according to the flow. Such classified traffic is less sensitive to communication between the D2D and C2D links, thereby reducing traffic overload. We evaluate the performance of the proposed scheme through simulation and show that the proposed scheme is more efficient than other comparison schemes.

## 1. Introduction

Fifth Generation (5G) is expected to rapidly change the age of mobile communication services by being applied to various applications such as automobile, media, security, life care, and energy industry. 5G is a very important communication technology that has an important influence on the Internet of Things (IoT) service, and 5G is expected to expand the application service category more than 4th Generation Long Term Evolution (4G LTE) mobile communications. Since the communication devices are expected to increase explosively in the age of 5G mobile communication, congestion control of traffic between Device-to-Device (D2D) User Equipments (DUEs) and Cellular User Equipments (CUEs) underlaying the 5G cellular network is an important issue to guarantee communication performance [1,2,3].

Cisco [4] expects to connect more than 50 billion communications devices and objects by 2020, and expects mobile traffics to increase by about 8.3 times compared to 2015. In the age of 5G mobile communication service, it is expected that explosive increase in the number of communication devices, so traffic interference control technology and high-performance service guarantee technology guaranteeing super-low latency and high reliability are very important technologies. Currently, the technology to achieve 5G performance goal in 5G wireless access networks is as follows.

- First, massive Multiple-Input and Multiple-Output (MIMO) technologies that increase spectral efficiency.
- Second, Multi-Carrier (MC) and Carrier Aggregation (CA) technology.
- Third, In-band Full Duplex (IFD) and Time Division Duplex (TDD) technology to maximize frequency utilization efficiency.
- Fourth, super high-frequency band utilization technology to provide wide bandwidth.
- Fifth, traffic interference control technology to effectively control large-sized traffic interference.
- Finally, small cell technologies to improve network capacity have been studied [5].

In particular, the multi-zone service technology underlying 5G cellular network can be applied to mid-haul technology through connection with many small cells, macro-cells and core networks, Remote Radio Unit (RRU) and Base Band Processing Unit (BBU) and a front-haul control technology between them. These technologies are technologies that improve multi-zone performance in 5G cellular networks and maximize service utilization. However, in order to guarantee 5G-based gigabit performance for these technologies, a technique to effectively control traffic problems occurring in these technologies is required [6,7,8].

Unlike the 5G multi-zone mobile communication service, the 4G multi-zone service includes D2D technology such as Zigbee, Bluetooth, WLAN, WiFi direct technology, and cellular approach such as Wideband Code Division Multiple Access (WCDMA)/LTE-A (Long Term Evolution Advanced). The communication technology of the mobile phone is mainly studied [9,10]. Large-sized traffic congestion control in multi-zone guarantees high data rate and low latency for User Equipments (UEs). Traffic congestion control and interference control techniques are important parameters to guarantee 5G performance. However, since the existing front-haul section is mainly used as a circuit-based interface and an optical exchange interface, it is difficult to efficiently control large-sized traffic as in the 5G network [5].

In order to solve this problem, various traffic interference control schemes based on resource allocations have been proposed in the 4G network. Wang et al. proposed a technique that combines power control and resource allocation techniques to control traffic interference between a cellular link and a D2D link [11]. Yin et al. proposed a distributed joint spectrum sharing and power allocation scheme to control traffic interference between DUEs underlaying a cellular network [12]. Duong et al. proposed a distance-based interference control scheme to control traffic interference caused by D2D communication underlaying cellular networks [13]. These techniques are useful for controlling traffic interference between a cellular link and a D2D link in a 4G cellular network environment, and it is difficult to efficiently control multiple interferences and congestion interference caused by large-scale traffic as in 5G. In particular, from the viewpoint of multi-zone service, large-sized traffic is transmitted to the communication between the cellular link and the D2D link. Therefore, detecting the traffic characteristic plays an important role in guaranteeing a high data rate and reducing the delay.

Most of all, the multi-zone service underlaying the 5G cellular network is affected by real-time traffic processes and non-real-time traffic processes, so detecting them adaptively is an important factor determining the performance of 5G set work. In a multi-zone service environment, if it cannot adequately control large-scale traffic, the 5G-based network link faces Quality of Service (QoS) issues such as latency, throughput degradation, and interference.

In this paper, we propose a new self-detection traffic interference control scheme, Self-detecting Traffic Interference Control Scheme (STICS), which detects large-sized traffic in real-time traffic processes and non-real-time traffic processes in order to reduce traffic interference between cellular links and D2D links in multi-zone networks. Real-time and non-real-time traffic processes are characterized by short-term traffic congestion processes and long-term traffic congestion processes, depending on the traffic characteristics. Here, real-time traffic processes are traffic processes that are less sensitive to delay, and conversely, non-real-time traffic processes are delay-sensitive traffic processes. Therefore, long-term traffic processes, which have a significant impact on traffic interference, are recorded in the post-recorder with low importance, and short-term traffic processes that hardly affect traffic interference are recorded in the pre-recorder. In this way, traffic processes managed by post-recorder and pre-recorder are easy to control traffic and increase overall traffic throughput.

The rest of this paper is as follows. Section 2 describes the related work. Section 3 describes the system model to guarantee multi-zone service. Section 4 describes the simulation analysis of the proposed method. Finally, we describe the conclusions.

## 2. Related Work

Recently, 5G-based cellular communications is a hot issue in wireless communication technology. 5G-based mobile communication has the goal of technology development to guarantee maximum transmission rate over 50 times and user transmission rate over 1Gbps compared with 4G. In order to guarantee 5G-based mobile communication performance, it is required to develop technologies such as wireless transmission technology that increases spectral efficiency, spectrum application technology with flexibility, technology that utilizes a super-high-frequency band, and compact cell technology [3,14]. These technologies are important technologies for guaranteeing the performance of 5G-based mobile communications, but unfortunately, these technologies have not yet established international standardization, and research is underway as a procedure for future 5G mobile communication services. Perhaps by 2022, several telecommunications service standardization techniques related to 5G-based mobile communications are expected to be presented in South Korea and other countries around the world. Mobile researchers expect large amounts of traffic to be generated between the cellular link and the device link when the 5G-based cellular network service environment is mature. This traffic will not only degrade the performance of 5G cellular communications services, but also will result in low data rates and high latency.

In order to solve these drawbacks, 4G cellular network has tried to solve them by applying the resource allocation solution between the cellular link and the D2D link. Cho et al. proposed a new spectrum sharing protocol for D2D communication overlaying a cellular network, which allows the D2D users to act as a relay to assist the two-way communications between the next Generation Node B (*gNB*) and *CUEs* [15]. Yu et al. proposed a resource-sharing optimization for D2D communication underlaying cellular networks, where *gNB* allocate either orthogonal or non-orthogonal radio resources to *CUEs* and DUEs [16]. Han et al. presented an uplink channel reusing selection optimization for D2D communication underlaying cellular networks, where an issue is an optimal and low-complexity channel reusing algorithm to maximize the number of admitted D2D pairs [10]. Lin et al. proposed the hybrid architecture that aims to improve the sum-rate and the power efficiency in 5G cellular networks.

The algorithm has been presented to maximize the system throughput while guaranteeing the QoS of both *CUEs* and DUEs [17]. Wen et al. proposed a QoS-aware mode selection and resource allocation scheme for D2D communication in the cellular network. The scheme analyzed the constraints and system equations of D2D links in scenarios with multiclass services, and it applied a joint mode selection and resource allocation (JMSRA) algorithm [18]. Zhang et al. proposed a resource allocation scheme for multi-D2D communications underlaying cellular networks with multi-subcarrier reusing [19]. The objective of this scheme is to improve the achievable rate of D2D communication and spectrum utilization, and it is to maximize the overall system spectrum efficiency while satisfying the rate requirements of all *CUEs* and guaranteeing that system gain is positive. However, the studies are done under the assumption that the direct communication mode is decided only by path loss without consideration of inter-user interference and requirements for communication quality. When a direct link cannot be supported in the D2D channel, DUEs and *CUEs* meet the service degradation.

Unlike 4G-based mobile communications, 5G-based mobile communications are very sensitive to traffic interference. In the age of 5G-based mobile communications, effective interference control due to large-sized traffic will ensure high data rates and improve service quality. In order to guarantee a high data rate and service quality in the 5G-based cellular network, several cooperative techniques are being studied. The cooperative schemes reduce the amount of overhead added on the back-haul and feedback channel underlying 5G cellular networks. CSI (channel state information) sharing cooperation scheme, MIMO cooperation scheme, and the relay-aided cooperative scheme is an important technique to reduce the heavy interference from adjacent cells. Therefore, multi-cell cooperation schemes are not only suitable but also necessary for 5G networks. However, these techniques cannot guarantee the same throughput for DUEs and *CUEs* in a multi-zone service environment, especially them does not detect a traffic interference source for inactive nodes and non-real-time traffic processes in which traffic is not performed and long-term traffic processes.

Therefore, in this paper, we propose to detect inactive nodes, real-time traffic processes and non-real-time traffic processes, and long-term traffic and short-term traffic in a timely manner under 5G-based cellular network environments.

## 3. System Model

**Figure 1:**
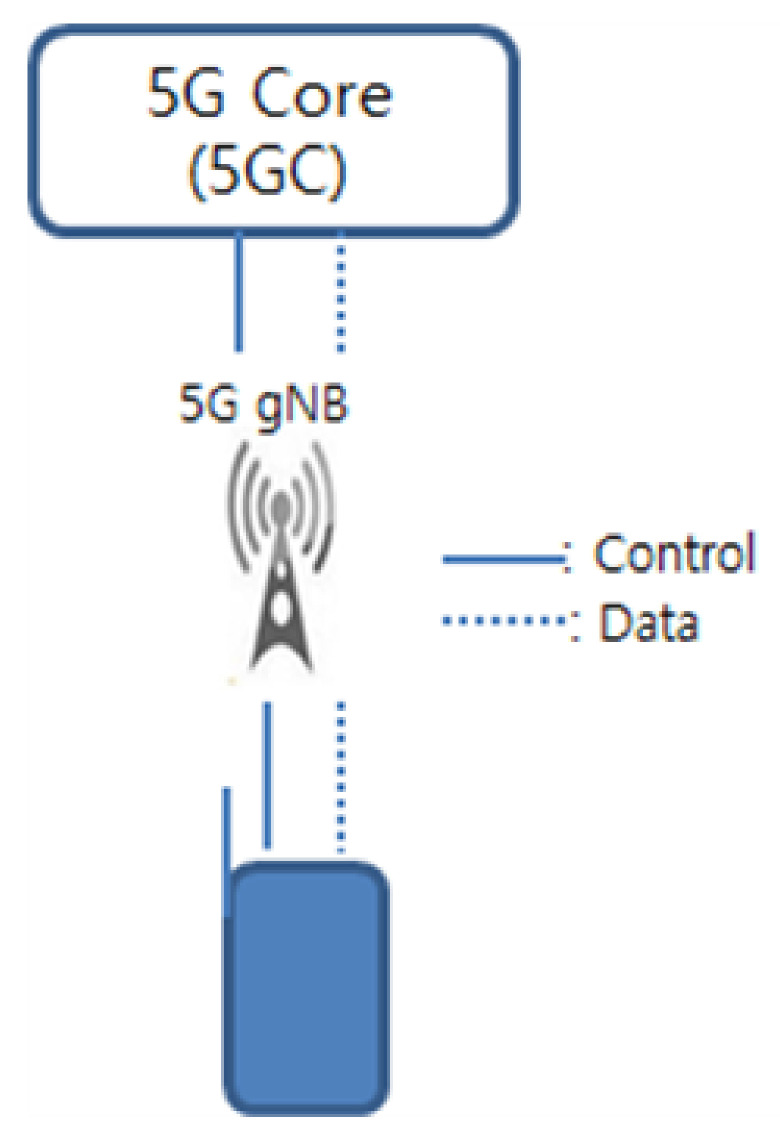
The basic structure of 5G next Generation Node B (*gNB*).

5G is called the 5th generation mobile communication technology, which focuses on the implementation of Enhanced Mobile Broad-Band (eMBB), Ultra-Reliable and Latency Communication (URLLC), and massive Machine Type Communication (mMTC) [20]. In 3rd Generation Partnership Project (3GPP), the 5G radio standard is called New Radio (NR), and the base station is called *gNB*. The core network is called 5G Core (5GC), and the basic structure of 5G *gNB* is shown in Figure 1.

**Figure 2:**
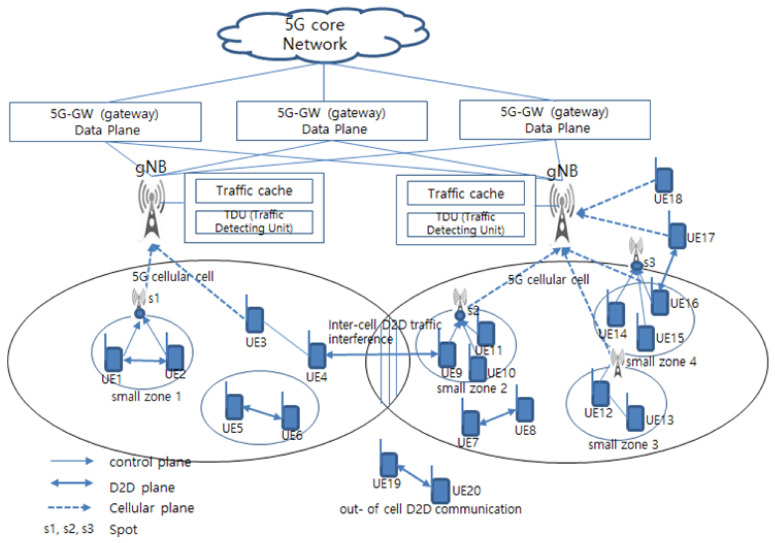
5G cellular network model.

In the 5G cellular networks, the multi-zone service is performed by the communication between the *gNB*, the UEs, and the UEs. In this paper, the proposed system model for self-detection traffic interference control is shown in Figure 2. The 5G core network in Figure 2 interfaces with 5G-GateWays (GW), and the 5G-GW interfaces with the *gNB* to perform 5G communications. The *gNB* is a base station that interfaces with the 5G-GW and detects and analyzes the small zone on the 5G cellular cell and the uplink traffic in each UEs.

The proposed multi-zone service consists of a communication terminal search process for searching small zones and D2D terminals capable of inter-terminal communication on 5G cellular cell, a link generation process for configuring a wireless link with *gNB*, small zone, *gNB*, *CUEs* and DUEs, and a traffic detection process for detecting whether interference occurs due to traffic when transmitting traffic in the process of generating a radio link. Link interference due to massive traffic congestion occurs when traffic is transmitted over a wireless link, and this massive traffic congestion provides the cause of severe performance degradation. In Figure 2, s1, s2, and s3 are spots where serious traffic is generated when a large amount of traffic is transmitted.

Therefore, in this section, we describe a technique to guarantee a 5G high data rate and communication service quality by self-detecting large-scale traffic at each spot.

### 3.1. Traffic Detecting Scheduler

The traffic-detecting scheduler first detects whether or not UEs are available for communication with UEs in their neighbors. At this time, the search for the nodes is performed by a TDU (Traffic Detection Unit). The TDU analyzes the cached traffic type and provides information about the traffic type to *gNB*. After that, the TDU detects uplink traffic in the link between the uplinked traffic and *gNB* and the *CUEs* in the small zone detect whether or not traffic interference occurs.

The TDU provides an important reference point for multi-zone service assurance, and it also monitors and analyzes traffic flows in the link between *gNB*, *CUEs* and CUEs, as well as uplinked traffic in the small zone. As shown in Figure 2, the *gNB* allows the TDU to monitor and analyze uplink traffic types from each small zone, and the traffic classification is handled by the scheduler.

**Figure 3:**
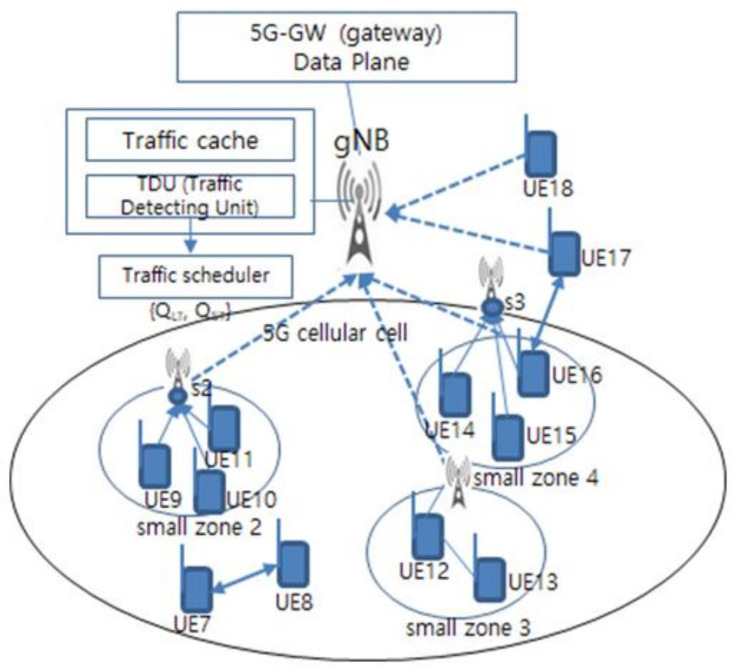
Traffic detecting scheduler.

The traffic scheduler detects whether the traffic type that is uplinked from the small zone is a large-sized traffic process such as video traffic or a small traffic process such as text and image. Figure 3 shows the process of detecting uplink traffic from small zones in the 5G cellular cell zone on the right side of Figure 2.

In Figure 3, $Q_{LT}$ is a queue that manages large traffic segments, and $Q_{ST}$ is a queue that manages the small traffic segment. The traffic scheduler is used to detect $Q_{LT}$ and $Q_{ST}$, and the detection of $Q_{LT}$ and $Q_{ST}$ in the traffic scheduler is performed by the scheduling metric λ. Here λ is a parameter that determines whether the type of traffic detected is a large traffic segment or a small traffic segment. 

Let us now assume that the uplinked traffic segments in the small zone of the 5G cellular cell zone are $ut_{1}^{}$, $ut_{2}$, …, $ut_{n}^{}$, and the time at which the traffic segments are bursting are $bt_{0}$, $bt_{1}$, …, $bt_{k-1}$. 

Let $bt_{i}$ be i^th^ traffic that is bursting. When the classification threshold for the i^th^ traffic is μ, the scheduling measures for each uplink traffic process are defined as follows.

**Definition** **1.** *Scheduling measure for uplink traffic process.*

(1) $$\lambda =\frac{1}{\sum_{i=1}^{n}(UT_{i}+TL_{i})+\sum_{j=1}^{k}\left(bt_{j}-bt_{j-1}\right)}\times \mu$$

Here *TL* is the size of the traffic segment and *bt* is the time at which the traffic segments are bursting. Therefore, in Definition 1, the scheduling metric *t* is affected by the correlation of the uplinked traffic process and the burst time, and the correlation directly affects the traffic detection and classification process.

### 3.2. Scheduling Strategy

The objective of the scheduling strategy is to determine when to schedule which uncached traffics so that the traffic interferences are reduced with the minimum amount of resource requirement. The following assumptions are included in the scheduling strategy.

- The traffic has been segmented and is transferred sequentially.
- The link spectrum of the *gNB*-*CUEs* is large enough to transfer the traffic to *gNB*.
- Each media object has its inherent encoding rate and traffic types.

The rate is not a constant in variable bit rate, but we apply $E_{R}$ to denote its average value. $AS_{gNB-CUE}$ may vary dynamically depending on their types when different traffic types are uplinked on *gNB*. PDU monitors $E_{R}$ by keeping records of data transmission rate of the most recent prior session. The transmission rate is calculated by detecting the amount of transferred traffic and traffic types during the transfer session. For the requested media traffics, assume there are n traffic segments cached in the 5G cellular cell. The objective of this process is to determine when to schedule traffic segment $TS_{n+1}$ so that the traffic interference is minimized. To accomplish the scheduling strategy, we suppose the notations are shown in Table 1.

When intuitively approaching, if $E_{R}<AS_{gNB-CUE}$, there is little traffic delay. However, if $E_{R}>AS_{gNB-CUE}$, then there is a delay due to a traffic explosion. This situation will then experience traffic interference, and as a result, this will degrade the performance of the 5G cellular network. 

To prevent traffic interference, the encoding rate of a certain traffic segment must overflow $AS_{gNB-CUE}$. Therefore, in order to avoid interference due to traffic explosion, the following conditions must be considered.

Condition 1: $\frac{\sum_{i=1}^{n}\left(TL_{i}-TL_{b}+TS_{n+1}\right)}{E_{r}}\geq AS_{gNB-CUE}$
Condition 2: $\frac{\sum_{i=1}^{n}\left(TL_{i}-TL_{b}+TS_{n+1}\right)}{E_{r}}\geq AS_{DUE_{i}-gNB}$

where $AS_{DUE_{i}-gNB}$ is the link channel from *gNB* to D2D link. 

If the above conditions are satisfied, the traffic detection strategy for each traffic is decided by $TL_{i}$ and $TL_{B}$, and we define the traffic detection metric P for the traffic detection strategy TDU is defined as follows.

**Definition** **2.** *Traffic detection metric P for TDU.*

(2) $$P=\sum_{i=1}^{n}TL_{i}-\frac{TS_{n+1}\times (E_{r}-AS_{gNB-CUE})}{AS_{gNB-CUE}}\times \mu$$

Thus, according to the traffic detection metric *P*, we consider a scheduling strategy to minimize traffic interference on the 5G cellular network as follows.

- with *n* = 0: In this case, the traffic segment is not cached on the *gNB* and traffic interferences are inevitable. To avoid future traffic interference, *gNB* is necessary to fetch the next ${\left\lceil \frac{E_{R}}{AS_{gNB-CUE}}\right\rceil }^{th}$ traffic segment. The minimum resource allocation required is $\left(1-\left(\frac{AS_{gNB-CUE}}{E_{R}}\right)\right)TL_{1}$.
- with n>0 and $(n+1)<\left(\frac{E_{R}}{AS_{gNB-CUE}^{}}\right)$: In this case, the *gNB* starts to cache the ${\left\lceil \frac{E_{R}}{AS_{gNB-CUE}}\right\rceil }^{th}$ traffic segment once the CUE starts to transfer the traffic segment. If the traffic segments between ${\left(n+1\right)}^{th}$ and ${\left\lceil \frac{E_{R}}{AS_{gNB-CUE}}-1\right\rceil }^{th}$ is transferred, they are sequentially cached, and it also meets the traffic interference. The minimum resource allocation required is equal to the resource allocation when *n* is zero.
- with *n* > 0 and $(n+1)\geq \left(\frac{E_{R}}{AS_{gNB-CUE}}\right)$: In this case, the caching of $TS_{n+1}$ starts to schedule when traffic segments reach the position of $\left((n+1)-\left(\frac{E_{R}}{AS_{gNB-CUE}}\right)\right)TL_{1}$. This case is not meet the traffic interference, and the minimum resource allocation required is $\left(1-\left(\frac{AS_{gNB-CUE}}{E_{R}}\right)\right)TL_{1}$.

In the 5G-based cellular network, this scheduling strategy provides the advantage of minimizing traffic interference while reflecting traffic types and network conditions for $Q_{LT}$ and $Q_{ST}$.

### 3.3. Interference Detecting Scheduler

Interference detecting scheduler for $Q_{LT}$ and $Q_{ST}$ is detected by considering the traffic transfer rate $T_{rate}$ and the packet throughput $T_{throughput}$. As shown in the scheduling strategy, if *n* = 0 or *n* > 0 and $(n+1)<\left(\frac{E_{R}}{AS_{gNB-CUE}}\right)$, *gNB* suffers from traffic interference due to uplink rates. On the other hand, if *n* > 0 and $(n+1)\geq \left(\frac{E_{R}}{AS_{gNB-CUE}}\right)$, traffic interference caused by cache overflow does not occur. This means that the traffic transmission is processing stably. However, if the traffic segments between (n+1)th and ${\left\lceil \frac{E_{R}}{AS_{gNB-CUE}}-1\right\rceil }^{th}$ are transferred, in this case, since $T_{throughput}$ is too low for $T_{rate}$, the traffic delay due to cache underflow is encountered. In order to solve this problem, we detect traffic interference type by considering $T_{rate}$ and $T_{throughput}$, and the detected traffic interference metric is defined as (3).

**Definition** **3.** *The detected traffic interference metric.*

(3) $$CT(i)=\frac{T_{rate}\times E_{R}}{T_{throughput}\times AS_{gNB-CUE}}$$

The *CUEs* and DUEs are sequentially forwarded to the *gNB*, and the traffic rate $T_{rate}$ at which the k^th^ traffic segment from the *CUEs* is transmitted to the *gNB* at an arbitrary time t is defined as Equation (4).

**Definition** **4.** *Traffic rate.*

(4) $$T_{rate}=\underset{k\to \infty }{\lim}\frac{1}{\sum_{i=1}^{k}\frac{t_{k}}{k}}$$

Here, the average traffic transmission rate is $\lambda^{-1}$, and the *gNB* periodically detects the average service rate by detecting Equation (4). Therefore, the traffic service rate C(α) based on $T_{rate}$ is defined as Equation (5).

**Definition** **5.** *Traffic service rate.*

(5) $$C(\alpha )=\frac{T_{throughput}-\left(\frac{1}{T_{rate}\times S_{time}}\right)\times \mu }{\left(1-\mu \right)}$$

Here μ is the traffic classification threshold and $S_{time}$ is the service time to service the transmitted traffic segment. C(α) is the traffic service rate satisfying the traffic threshold. If *n* > 0 and $(n+1)<\left(\frac{E_{R}}{AS_{gNB-CUE}^{}}\right)$ in the interference detection process, it meets $Q_{LT}$ state. In this case, the traffic transmission state falls into the long-term congestion state, resulting in a traffic delay. 

However, if the *gNB* is $(n+1)\geq \left(\frac{E_{R}}{AS_{gNB-CUE}}\right)$, the traffic state is transformed into a stable traffic transmission mode, and in this case, the *gNB* does not meet the interference. Thus, the interference detection procedure is an important metric to measure traffic transmission state and link congestion state for *gNB*, DUEs and CUEs, and it is also an important metric to guarantee gigabit communication.

### 3.4. Traffic Interference Control

In 5G-based cellular networks, real-time application traffic is sensitive to delay, and these delays require strict QoS. Therefore, in this paper, it is assumed that retransmission due to high-capacity real-time traffic delay is not allowed. If the traffic segments that DUEs and *CUEs* request for uplink cause link congestion, the *gNB* will meet traffic segment processing delays caused by data overflow. The interference control procedure detects the delay by considering traffic segment delay, long-term and short-term propagation delays between CUE and *gNB*, long-term and short-term propagation delay between DUEs and *gNB*, and throughput for real-time and non-real-time application traffic. Therefore, we define the queuing delay that occurs when a traffic segment is transmitted from an arbitrary $CUE_{i}$ to *gNB* as Equation (6).

**Definition** **6.** *Queueing delay.*

(6) $$D_{RTTS}=\frac{R_{RTTS}}{T_{RTTS}}$$

The link queuing delay that occurs when any $DUE_{i}$ and $CUE_{i}$ reach *gNB* from the source path is defined as Equation (7).

**Definition** **7.** *Link queueing delay.*

(7) $$D_{q}=\sum_{i,\sin k\in path}\frac{R_{RTTS}}{T_{RTTS}}$$

Then, the propagation delay for the arrival of any $DUE_{i}$ and $CUE_{i}$ from the base path to *gNB* is defined as Equations (8) and (9).

**Definition** **8.** *Propagation delay from CUE_i_ to gNB.*

(8) $$PD_{CUE-gNB}=\sum_{CUE-gNB\in link}\frac{R_{RTTS}}{T_{RTTS}^{}}+\sum_{CUE-gNB\in link}\lambda \times Dist_{CUE-gNB}$$

**Definition** **9.** *Propagation delay from gNB to DUE_i_.*

(9) $$PD_{DUE-gNB}=\sum_{DUE-gNB\in link}\frac{R_{RTTS}}{T_{RTTS}^{}}+\sum_{DUE-gNB\in link}\lambda \times Dist_{DUE-gNB}$$

Here $Dist_{CUE-gNB}$ and $Dist_{DUE-gNB}$ is the distance from $DUE_{i}$ and $CUE_{i}$ to *gNB*, respectively, and λ is the scheduling metric. We also consider interference control on the *gNB* simultaneously to control interference to traffic segments. Therefore, the SINR between *gNB* and $CUE_{i}$ is defined as Equation (10), and the Signal to Interference plus Noise Ratio (SINR) is measured by considering both interference and delay on the 5G-based cellular network.

**Definition** **10.** *SINR.*

(10) $$gNB_{SINR}-\frac{{\left|E_{R}^{}\right|}^{2}Dist_{CUE-gNB}\times T_{rate}}{{\left|AS_{CUE-gNB}\right|}^{2}\times T_{throughput}+S(i)}$$

Thus, we control the interference to minimize the delay due to the traffic segment transmission, and when $(n+1)\geq \left(\frac{E_{R}}{AS_{gNB-CUE}}\right)$, the SINR at the *gNB* is minimized. Therefore, the proposed traffic interference control scheme provides the advantage of reducing the interference burden on the $CUE_{i}$ transmission by ensuring maximum data transmission to the *gNB*.

### 3.5. STICS Procedure

In 5G-based cellular networks, the operation to control interference caused by high traffic rates and neighboring radio resources is operated by the STICS procedure, and this mechanism schedules a scheduling mode for 5G-based cellular network services. In the STICS procedure, communication between UEs is divided into two types: CUE and DUE, CUE is users who perform cellular communication in the cellular mode of *gNB*, and DUE is users who directly perform D2D communication. In the STICS procedure, if the scheduler for traffic interference management does not have performance parameters such as neighboring radio channels, SINR, traffic rate, and propagation delay, the cellular link suffers from interference. To mitigate this overhead, the STICS procedure operates an algorithm, and Algorithm 1 shows the STICS procedure.

**Algorithm 1:** STICS procedure.Step1: $R_{QUEUE}$: Resource allocation queue for UEsStep2: $RB_{n}$: Resource blocks assigned to *gNB*Step3: $UE_{set}$: *CUEs* and DUEs waiting for resource assignment and traffic rate Step4: Calculate CT(i), C(α), $PD_{CUE-gNB}$, $PD_{DUE-gNB}$, and SINR
Step5: while $R_{QUEUE}$≠0 and $UE_{set}$≠0 doStep6: For all $R_{QUEUE}$∈$UE_{set}$ doStep7: Calculate C(α) and SINR
Step8: end forStep9: Assign $RB_{n}$ satisfying C(α) and SINR to *CUEs* and DUESStep10: whileStep11: if $UE_{set}$≠0 thenStep12: $CUEs\leftarrow \frac{CUE}{UE_{set}},\;DUEs\leftarrow \frac{DUE}{UE_{set}}$
Step13: endStep14: OtherwiseStep15: begin Step16: Go to Step4Step17: end 

The scheduler in the STICS procedure of Algorithm 1 measures equations such as the traffic interference metric in Equation (3), the traffic service rate in Equation (5), the propagation delay in Equations (8) and (9), and SINR in Equation (10) to allocate radio resources to *CUEs* and DUEs, and therefore it does not suffer from traffic interference. After processing such an operation, it schedules the resource allocation queue, and the purpose of scheduling the resource allocation queue is to reduce the latency time due to resource allocation. As shown in Algorithm 1, it can be seen that SUEs effectively allocate resources to *CUEs* and DUEs by the STICS procedure, and thus *CUEs* and DUEs stably control traffic interference by Algorithm 1.

## 4. Simulation Results

In this section, we analyze average traffic throughput and average propagation delay to investigate the performance of the proposed scheme. The average propagation delay is the average delay time for any DUEi and CUEi to reach the *gNB* from the base path. We selected the 5G cellular network structure shown in Figure 2 as the domain for the simulation and set the zone radius to 200 m. In each zone, it is assumed that *CUEs* and DUEs are uniformly distributed. We evaluate and analyze the performance of 5G cellular network structure considering only *t*, and simulation parameters are shown in Table 2. 

We used the CSMA (Carrier Sense Multiple Access)/MAC (Media Access Control) protocol for 5G cellular network structures to provide fair upload opportunities for DUEs and CUEs. We assumed that DUEs and *CUEs* are uniformly distributed on the 5G cellular network in Figure As shown in Figure 2, we measured the overflow detection rate and traffic throughput when μ≥0.7 and N = 200,000 to investigate the performance of the proposed scheme in a virtual 5G cellular network. We set μ to a greater value than 0.7 because μ generated the worst-case congestion overflow when μ is less than 0.7. Table 3 shows the experimental results for overflow detection rate and traffic throughput when μ≥0.7 and N = 200,000. We compared non-STICS, pairing, and performance with the proposed STICS.

As shown in Table 3, the proposed STICS algorithm showed that the overflow detection rate is improved about twice as much as the non-STICS algorithm and the performance was improved by about 20% than the paring method. We measured traffic service rate and propagation delay by applying traffic threshold μ and the number of traffic segments N. Traffic classification thresholds were classified into three levels: Low, Medium, and High. We set μ≤0.3 for Low, 0.4<μ<0.7 for Medium and μ≥0.7 for High, respectively. We classified the traffic threshold μ into three levels: Low, Medium, and High. The reason for this is to investigate the correlation between the traffic classification threshold and the traffic service rate and propagation delay. 

**Figure 4:**
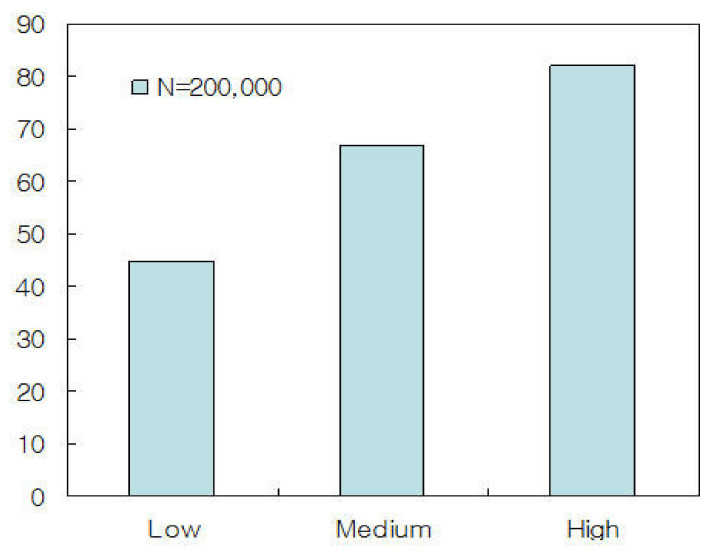
Traffic service rate for low, medium, and high when the number of traffic segments is N = 200,000.

Figure 4 shows traffic service rates for Low, Medium, and High when the number of traffic segments is N = 200,000. As shown in Figure 4, it can be seen that the traffic service rate is high when the traffic classification threshold is low in the *gNB*. On the contrary, if the traffic classification threshold is large, the traffic service rate is low. As a result, it can be seen that the traffic service rate is affected by the traffic classification threshold. Therefore, if the TDU detects traffic in a timely manner, the proposed STICS will be guaranteed gigabit communication services.

**Figure 5:**
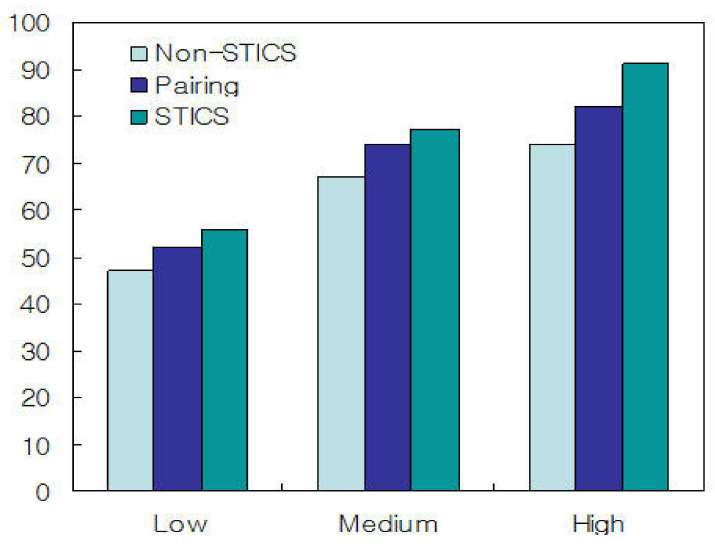
Average traffic service rate for non-Self-detecting Traffic Interference Control Scheme (STICS), pairing, and STICS when μ is low, medium, and high.

Figure 5 shows the average traffic service rate for non-STICS, pairing and proposed STICS when μ is Low, Medium, and High. As shown in Figure 5, the proposed STICS algorithm is superior to other algorithms for traffic service rate. This is because the proposed algorithm efficiently detects traffic interference and controls overflow errors according to the traffic type in real-time. 

Figure 5 shows the average propagation delay rate when N = 200,000. To simulate the propagation delay rate, we classified traffic segments into 5 groups: G1, G2, G3, G4, and G5, and randomly allocated traffic segments to each group. As shown in Figure 5, the propagation delay rate is affected by the threshold μ, and when the threshold μ is high, it can be seen that the average propagation delay rate is optimized.

**Figure 6:**
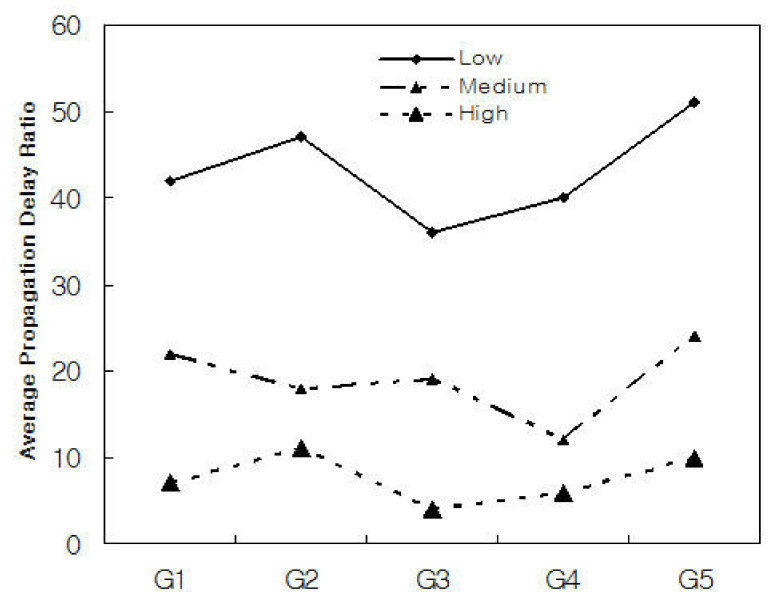
Average propagation delay ratio for each group with low, medium, and high.

**Figure 7:**
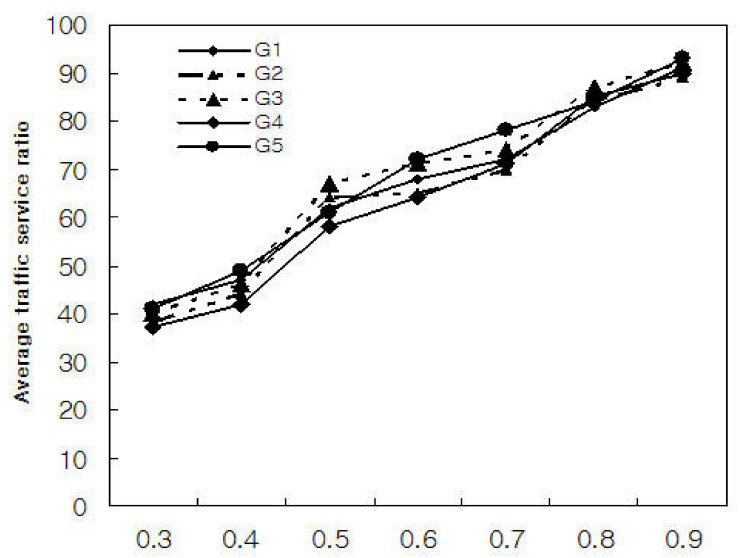
Average traffic service ratio for each group with μ.

Figure 6 and Figure 7 show the experimental results of applying the traffic threshold μ to each group by dividing N = 200,000 into 5 groups. We classified the five groups into G1, G2, G3, G4, and G5, and assumed that each group is randomly assigned real-time and non-real-time and long-term and short-term application traffic processes. We randomly assigned 40,000 different traffic types to each group and performed traffic transmission five times.

As shown in Figure 7, it can be seen that the traffic threshold μ affects propagation delay and traffic services. As shown in the figure, when the traffic threshold μ is small, the average traffic service ratio for each group showed low experimental results, and conversely, it showed improved traffic service rate when it is large. This means that the traffic threshold μ is affected by the scheduling metric λ. As a result, it can be seen that the performance of the 5G network is affected by the traffic control algorithm.

Therefore, when communication between DUEs and *gNB*, and communication between *CUEs* and *gNB* in the 5G cellular network are not controlled in a timely manner, the throughput of traffic decreases. This means that gigabit communication becomes more difficult. We experimentally analyzed the propagation delays and the causes of service degradation between DUEs, *gNB*, and *CUEs* and *gNB* to solve these problems and it ensures gigabit communication services. The results showed that the propagation delay is affected by the traffic overflow and that the traffic overflow is affected by congestion. 

In South Korea, major telecommunication companies such as SK Telecom, KT Telecom, and LGT are actively providing 5G mobile communication services to users. Despite these 5G mobile communication services, users suffer from unstable types of services due to high traffic rates. The proposed mechanism is expected to be free from such service drawbacks, and this mechanism will be also applied to 5G application domains such as smart farm, smart factory, IoT (Internet of Things), and transport industry, resulting from high traffic rates, and it is expected to be able to effectively manage and control the interference type due to high traffic rates.

In the future, the authors will continue to study how to minimize traffic interference by applying the proposed algorithm to real 5G cellular network environment, and the proposed mechanism expects that the scheduler algorithm will contribute to future 5G based mobile communication service. 

## 5. Conclusions

In a 5G-based cellular network environment, multi-zone services are severely affected by wireless traffic. Wireless traffic causes resource interference, and resource interference makes gigabit performance difficult to guarantee and reduces traffic throughput. In this paper, we proposed a self-detecting traffic interference control scheme (STICS) to minimize the overflow problem caused by traffic interference when DUEs and *CUEs* communicate with *gNB* in a multi-zone service environment. In order to minimize traffic interference between the cellular link and the D2D link, the proposed scheme detects and controls whether the traffic is a real-time traffic type or a non-real-time traffic type. 

The traffic control procedure is based on interference detection, and it processes a traffic operation through detection of queuing delay and propagation delay in transmission of traffic segments from DUEs and *CUEs* to *gNB*. Simulation results showed that the proposed scheme is superior to the Non-STICS scheme and pairing scheme in overflow and traffic processing, and also effectively controls the propagation delay. The performance of the proposed scheme was about twice as high as that of Non-STICS, and the average throughput was about 30%. Future research will focus on 5G-based cellular systems to guarantee gigabit services and performance improvement techniques to guarantee gigabit services by applying the proposed method to the 5G platform. If standardization of 5G-based mobile communication service development is set up in International Telecommunication Union-Radiotelecommunication (ITU-R), we will contribute to the development of practical 5G-based mobile communication service by applying the proposed technique to 5G mobile communication environment.

## Figures and Tables

**Table 1:** Notations for scheduling strategy.

Notations	Descriptions
$E_{R}$	The average encoding rate of a traffic segment
$AS_{gNB-CUE}$	The average spectrum of gNB-DUEs link
N	The total number of traffic segments of gNB-DUEs link
n	The number of cached traffic segments on the *gNB*
$bt_{j}$	The bursting time for traffic segments
$TL_{i}$	The length of the *i*th traffic segment
$TL_{B}$	The original length for traffic segments
$TS_{n+1}$	The Traffic Segment
μ	Traffic threshold
$D_{P}$	Propagation delay between *CUEs* and *gNB*
$R_{RTTS}$	The transmission rate for the real-time traffic segment
$D_{RTTS}$	Delay rate for real-time traffic segment
$T_{RTTS}$	Throughput for real-time traffic segment

**Table 2:** Simulation parameters.

Parameters	Values
$E_{R}$	1–10 Mbps
$AS_{gNB-CUE}$	Dual 500 Mbps
N	200,000
n	120,000
$bt_{i}^{}$	0.1 ms
$TL_{i}^{}$	7 Mbps
$TL_{b}^{}$	5 Mbps
μ	0≤μ≤1
λ	0 < λ < 1
SINR	10–15 dB

**Table 3:** Performance for comparison algorithm. with μ≥0.7 and N = 200,000.

Algorithms	Non-STICS	Pairing	STICS
Overflow Detection Rate	26	42	53
Traffic Throughput	62	87	94

## Data Availability

Data sharing not applicable.
